# Adjunctive Use of Ozone Therapy in Treating Periodontitis in Smokers: A Prospective Split‐Mouth Study

**DOI:** 10.1155/ijod/6693225

**Published:** 2026-06-03

**Authors:** Hanadi Ghurmallah Alzahrani, Banna Alnufaiy, Hassan Alkharaan, Abdulaziz Alsakr, Faisal Mohammed Alharbi, Naif Salem Alanazi, Muath Abdullah Alsuwailem, Khalid Gufran

**Affiliations:** ^1^ Department of Preventive Dental Sciences, College of Dentistry, Prince Sattam bin Abdulaziz University, Alkharj, 11942, Saudi Arabia, psau.edu.sa; ^2^ Dental Intern, College of Dentistry, Prince Sattam bin Abdulaziz University, Alkharj, 11942, Saudi Arabia, psau.edu.sa

**Keywords:** ozone therapy, periodontitis, smokers, stages and grades of periodontitis

## Abstract

**Objective:**

To evaluate the short‐term effectiveness of adjunctive ozone therapy combined with scaling and root planing (SRP) in smokers with Stages II and III periodontitis.

**Methods:**

This prospective randomized split‐mouth study included 26 smokers with Stages II or III periodontitis. In each participant, one side received SRP alone (control) and the contralateral side received SRP plus ozone therapy (test). Probing depth (PD), clinical attachment loss (CAL), bleeding on probing (BOP), and plaque index (PI) were recorded at baseline and 6 weeks. Paired *t*‐tests were initially used then more appropriately account for the split‐mouth design and repeated measurements within the same participant, linear mixed‐effects models were then fitted. Adjusted models additionally included age, periodontitis stage, smoking duration, and smoking.

**Results:**

The mean age of participants was 35.23 ± 8.64 years, and the mean number of teeth was 26.76 ± 4.50. All clinical parameters improved after treatment. In mixed‐model analyses, baseline values were comparable between study and control sides. In the adjusted model, the ozone‐treated side showed significantly greater additional improvement than the control side for PD (*β* = −0.346 mm, 95% CI: −0.633 to −0.059; *p* = 0.019), CAL (*β* = −0.462 mm, 95% CI: −0.713 to −0.211; *p*  < 0.001), BOP (*β* = −0.063, 95% CI: −0.108 to −0.018; *p* = 0.007), and PI (*β* = −0.077, 95% CI: −0.129 to −0.025; *p* = 0.004).

**Conclusion:**

The adjunctive use of ozonated water with SRP was associated with greater short‐term improvements in periodontal clinical parameters compared with SRP alone in smokers with Stages II and III periodontitis.

**Clinical Relevance:**

Smoker’s experience severe forms of periodontitis. Ozone demonstrated positive effects in reducing plaque‐related outcomes when used as an adjunct to SRP.

## 1. Introduction

Periodontitis is a persistent inflammatory condition involving multiple factors that are often linked to an imbalance in plaque biofilms [1], with disease gradually breaking down the structures that support the teeth. Its main features include loss of support for periodontal tissues, which can be visualized as clinical attachment loss (CAL) and alveolar bone loss measured on radiographs, as well as the presence of periodontal pockets and bleeding gingiva [1]. Considering the pathophysiological mechanisms involved, researchers have identified three distinct categories of periodontitis as manifestations of systemic disease periodontitis, necrotizing periodontitis, and chronic periodontitis [2].

Periodontal disease is a chronic infectious and inflammatory condition that affects susceptible individuals and is a leading cause of tooth loss in adults worldwide [3]. Cigarette smoking is a global public health concern, has a detrimental impact on both general and oral health, and significantly increases the risk for periodontal disease and oral cancer [4, 5]. Epidemiological research has shown that smokers are at a significantly higher risk for developing periodontal disease than nonsmokers, with the risk correlating directly with the duration and intensity of smoking [6, 7].

Clinically, smokers tend to demonstrate a distinct susceptibility to periodontal disease, exhibiting deeper periodontal pockets, more advanced CAL, greater bone destruction, and a higher incidence of tooth loss [8]. Chronic tobacco use is associated with increased gingival microvascular density due to enhanced capillary recruitment. However, these capillaries are often more tortuous and narrower. These structural changes, combined with repeated vasoconstriction from smoking, result in reduced gingival blood flow that does not fully recover, even after smoking cessation. Gingival inflammation and angiogenesis are typically noticeable in nonsmokers with periodontal disease, both of which are significantly suppressed in chronic smokers due to local immune suppression and oxidative stress. Regardless of the form in which the tobacco is used, prolonged exposure leads to microvascular dysfunction, increasing the risk for disease progression and complications related to treatment [9].

Periodontal therapy consists of three phases: nonsurgical, surgical, and supportive care [10, 11]. The success of treatment depends heavily on collaboration between the patient and clinician [12]. Additionally, systemic health, environmental exposure, and psychological stress can also influence treatment outcomes [1, 13]. Research indicates that both nonsurgical and surgical periodontal treatments tend to be less effective in smokers than in nonsmokers [13]. Smoking is now recognized to be a major environmental risk factor for periodontal disease. This understanding has been incorporated into the current periodontal disease classification system, in which the grade of periodontitis is adjusted based on smoking status and the number of cigarettes smoked daily [1, 13]. The primary objective of periodontal treatment is to eliminate or significantly reduce pathogenic bacterial load and associated inflammation, leading to shallower probing depth (PD) and improved CAL. Scaling and root planing (SRP) is the most widely used nonsurgical approach [14]; however, smokers generally exhibit a less favorable response to SRP than nonsmokers. Several clinical studies have reported smaller reductions in PD and fewer improvements in CAL in smokers after SRP [15, 16]. Conversely, other studies have reported comparable clinical outcomes between smokers and nonsmokers after nonsurgical treatment [17, 18].

Various adjunctive therapies, including topical antiseptics and systemic or local antibiotics, have been explored to enhance the efficacy of SRP in the management of periodontal diseases [19]. However, findings regarding the effectiveness of these adjunctive treatments remain inconsistent, with no universal consensus regarding the optimal approach to complement mechanical therapy [20, 21].

Ozone therapy is gaining attention due to its strong antimicrobial properties. It is effective against a broad spectrum of microorganisms, including both gram‐positive and gram‐negative bacteria, as well as against resistant strains such as *Pseudomonas aeruginosa* and *Escherichia coli*. Ozone also demonstrates antiviral, antifungal, and sporicidal activity [22]. As an unstable triatomic molecule naturally found in the stratosphere, ozone decomposes quickly to release nascent oxygen, making it a potent oxidizing agent an attribute that lends it self to medical and dental applications. Its high oxidation potential makes ozone particularly useful for periodontics and other dental treatments [23].

In our literature review, we identified a knowledge gap in which only one study assessed the combined use of ozone therapy and scaling in both smokers and nonsmokers with gingivitis but not periodontitis and reported significant improvements in clinical parameters with ozone use alongside periodontal therapy [24]. To date, no study has specifically evaluated the effects of adjunctive ozone therapy in smokers with periodontitis. As such, this study aimed to assess the effectiveness of ozone therapy as an adjunct to conventional nonsurgical periodontal treatment in smokers diagnosed with Stages II and III periodontitis by measuring PD, CAL, bleeding on probing (BOP), and O’Leary plaque index (PI) before and after treatment. We hypothesized that ozone therapy would be an effective adjunctive therapy for smokers diagnosed with periodontitis.

## 2. Materials and Methods

### 2.1. Study Design and Population

This prospective study was conducted at the College of Dentistry at Prince Sattam Bin Abdulaziz University (Al‐Kharj, Saudi Arabia). Ethics approval was obtained from the Standing Committee of Bioethics Research (SCBR) of Prince Sattam bin Abdulaziz University (SCBR‐158/2023), and informed consent was obtained from all participants.

### 2.2. Sample Size Estimation

The sample size estimation was performed for the primary variable, CAL, using the following equation:

n=fα,β×σ2/μ1−μ22.

Based on this calculation, a minimum sample size of 20 participants was determined, assuming a clinically significant difference (0.5 mm in CAL between groups, a standard deviation of 0.7, 80% power, and a significance level [i.e., alpha] of 0.05). To account for an anticipated 20% loss to follow‐up by the end of the treatment period, the final sample size was increased to 26. These estimates were used in split‐mouth literature such as a randomized periodontal trial by Park et al. [25] likewise stated that 21 patients would detect a 0.5 mm between‐treatment difference in PD and CAL change with 80% power and SD 0.7 mm.

### 2.3. Eligibility and Group Allocation Process

Participants attended a screening visit in the periodontics and interns’ clinic, during which they underwent a clinical examination and completed a data collection form capturing information such as age, smoking status, baseline clinical parameters, and periodontal diagnosis. Eligible participants were active smokers aged 20–70 years, diagnosed with generalized periodontitis (Stages II or III) according to the 2017 World Workshop classification [1], and had ≥12 teeth evenly distributed across all quadrants of the mouth. Smoking status was confirmed during the screening visit and again at the follow‐up appointment. Participants received routine advice regarding smoking cessation; however, no structured cessation program was implemented as part of the study protocol. The exclusion criteria included individuals with diabetes, pregnant or lactating women, those who had undergone periodontal therapy or received antibiotics within the previous 3 months, and those undergoing chemotherapy.

In this split‐mouth study design, each participant served as their own control. Randomization was performed using a simple randomization method by an independent individual who was not involved in the clinical procedures or outcome assessment. During the initial visit, the test intervention (ozone therapy) was randomly assigned to either the left or right side of the mouth, while the contralateral side received SRP alone as the control treatment. The allocation was recorded on a data collection form and placed in a sealed envelope to ensure allocation concealment.

**Figure 1:**
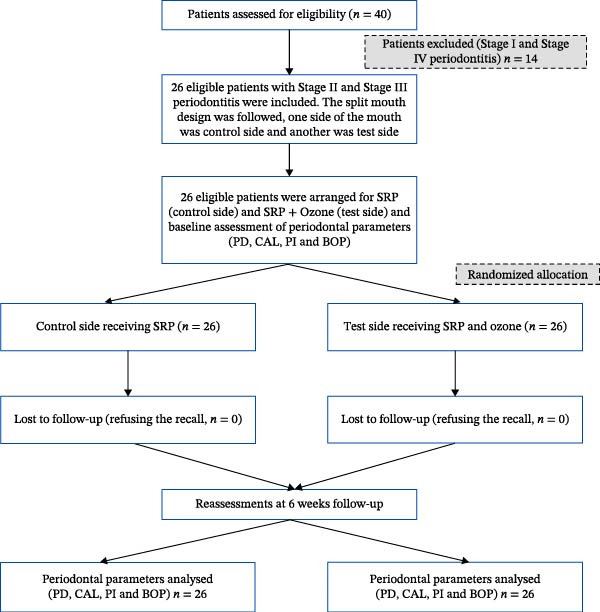
Flow chart of study participants.

At the 6‐week follow‐up visit, clinical measurements were recorded by a blinded examiner using a new data collection form that identified only the left and right sides, without indicating which side had received ozone therapy. After completion of data collection, the sealed envelopes containing the allocation information were opened and the data were provided to the statistician for analysis. A CONSORT flow diagram illustrating participant recruitment, allocation, follow‐up, and analysis is presented in Figure 1.

### 2.4. Clinical Examination

Baseline periodontal measurements were recorded prior to any professional supragingival scaling to document the untreated periodontal condition of the participants. Clinical measurements, including PD, CAL, BOP, and O’Leary PI, were documented at baseline and at 6 weeks after treatment by a single blinded examiner to ensure measurement consistency. All participants received standardized oral hygiene instructions, including guidance on proper tooth brushing technique and plaque control measures at baseline. Oral hygiene instructions were reinforced during the follow‐up visit to encourage maintenance of adequate plaque control throughout the study period.

Full‐mouth PD evaluation used the UNC 15 probe, inserted parallel to the tooth’s long axis, to assess the deepest point of the pockets at six sides per tooth, excluding third molars. The distance from the gingival margin to the base of the pocket was recorded, and the maximum PD was recorded for each patient. CAL measurements from the cementoenamel junction to the apical end were performed using the same probe, with the highest value among the CAL measurements documented for each patient.

Additionally, BOP was assessed by gently inserting a periodontal probe into the sulcus and sweeping through the proximal surface; any observed bleeding after 30 s indicated a positive BOP [26]. PI evaluation involved chewing a discoloring agent tablet, ensuring that the mixture coated all tooth surfaces, and electronically recording plaque presence in the periodontal chart based on the observed discoloration. PI was calculated using an equation considering all tooth surfaces. Teeth were stained using a disclosing solution (presence of plaque) divided by the number of surfaces examined [27].

### 2.5. Intervention

In each patient, two quadrants with ≥3 teeth with an interdental CAL ≥3 mm were randomly assigned to either the test group (SRP combined with ozone therapy [*n* = 26]) or the control group (SRP alone [*n* = 26]). Local anesthesia preceded SRP, which involved the use of Gracey and universal curettes along with ultrasonic instruments, with a minimum of 20 strokes per tooth during a single visit. The Apruio3 machine (Model: APL‐OTM‐3125, Shenzhen, China ‐518055) was used to obtain ozone. The freshly generated ozone water (5–20 μg/mL) was applied once immediately after SRP for approximately 5–10 min per site using freshly generated ozonated water delivered intrasulcularly. For irrigation, a 2 mL syringe with a 24‐gauze needle was utilized. The needle was bent in the center at an angle of 110° before being inserted intrasulcularly.

The freshly generated ozone water (5–20 μg/mL) was used based on manufacturer’s instructions.

### 2.6. Blinding and Calibration

A single trained and calibrated investigator recorded the PI, PD, CAL, and BOP measurements. The clinical examiner who recorded the periodontal measurements was blinded to the treatment allocation. The examiner was calibrated by experts for plaque and periodontal assessment using these indices. Intraexaminer reliability was assessed using kappa statistics, which were 0.80 for periodontal evaluations and 0.84 for the PI.

### 2.7. Statistical Analysis

Data collected in the present study were analyzed using SPSS version 20 (IBM Corp., Armonk, NY, USA). Descriptive statistics, including mean, standard deviation, frequency, and percentage, were computed to summarize the demographic and outcome variables. The Shapiro–Wilk test was used to assess data normality, which helped guide subsequent statistical analyses. A paired *t*‐test was performed to compare outcome variables between the test and control groups at baseline and at week 6. A paired *t*‐test was used for both between‐group and within‐group comparisons because the measurements were not independent and each patient acted as their own control. Differences with *p* < 0.05 were considered to be statistically significant. Data were also analyzed using a linear mixed‐effects modeling approach to account for the split‐mouth design and repeated measurements within the same participant. For each outcome variable, including PD, CAL, BOP, and PI, a primary mixed model was fitted with treatment side (control vs. study), time (baseline vs. 6 weeks), and the treatment‐by‐time interaction as fixed effects, with patient‐level random effects used to account for within‐subject correlation between repeated observations from the same individual and between mouth sides within the same participant when supported by the data structure. The treatment‐by‐time interaction was considered the main parameter of interest, as it represented the additional change in the study side beyond that observed in the control side. A secondary adjusted mixed model was then fitted for sensitivity analysis by additionally including age, periodontitis stage, smoking duration, and number of cigarettes smoked per day as fixed covariates. Model estimates were reported as regression coefficients (*β*) with 95% confidence intervals and *p*‐values. Model fit was evaluated using Akaike information criterion (AIC), Bayesian information criterion (BIC), and marginal and conditional *R* ^2^ values. A two‐sided *p*‐value <0.05 was considered statistically significant.

## 3. Results

A total of 26 participants were selected for the present split‐mouth investigation (26 side control–26 side test); baseline characteristics are summarized in Table 1. The mean age of the participants was 35.23 ± 8.64 years, and the mean number of teeth was 26.76 ± 4.50. Most participants (61.5%) were cigarette smokers, with a mean cigarette pack‐year history of 15.57 ± 3.33. The mean duration of tobacco use was 13.26 ± 8.45 years. Approximately 53.8% of participants were diagnosed with Stage II periodontitis, one‐half of whom were classified as having Grade II periodontal disease. The clinical parameters of the study participants at the test side before and after the intervention are summarized in Table 2. The mean BOP score decreased significantly from baseline (75.26 ± 13.19) to the week 6 of intervention (55.92 ± 9.34), with a statistically significant difference (*p* = 0.000). There was also a substantial reduction in PI score from baseline to postintervention (82.86 ± 13.80 vs. 61.23 ± 13.47; *p* = 0.000). The mean CAL exhibited a statistically significant decrease from baseline (4.84 ± 1.37) to week 6 (4.15 ± 1.43; *p* = 0.000). The mean pocket depth was also reduced significantly from baseline (4.53 ± 0.98) to week 6 (3.80 ± 0.93; *p* = 0.000).

**Table 1:** Baseline characteristics of study participants.

Characteristics	Mean ± SD
Age (years)	35.23 ± 8.64
Number of teeth present	26.76 ± 4.50
Duration of tobacco consumption in years	13.26 ± 8.45
Cigarette pack per years	15.57 ± 3.33
Type of tobacco consumption	*N* (%)
Hookah	3 (11.5%)
Cigarette	16 (61.5%)
Vape	4 (15.4%)
Chewing tobacco	2 (7.7%)
Former tobacco user	1 (3.8%)
Stage of diagnosis	
Stage II	14 (53.8%)
Stage III	12 (46.2%)
Grade	
Grade II	13 (50%)
Grade III	13 (50%)

*Note: N*, number of participants.

Abbreviation: SD, standard deviation.

**Table 2:** Within group comparison of clinical parameters before and after the intervention in both test and control sides.

Variables	*N*	Test side				Control side			
		Baseline (mean ± SD)	6^th^ week (mean ± SD)	95% CI	*p*	Baseline (mean ± SD)	6^th^ week (mean ± SD)	95% CI	*p*
BOP	26	75.26 ± 13.19	55.92 ± 9.34	15.50, 23.18	0.000	75.33 ± 13.02	62.30 ± 10.73	9.38, 16.66	0.000
PI	26	82.86 ± 13.80	61.23 ± 13.47	16.69, 26.57	0.000	82.86 ± 13.80	68.88 ± 12.38	10.35, 17.60	0.000
CAL	26	4.84 ± 1.37	4.15 ± 1.43	0.50, 0.88	0.000	4.88 ± 1.55	4.65 ± 1.69	0.05, 0.40	0.011
PD	26	4.53 ± 0.98	3.80 ± 0.93	0.5, 0.941	0.000	4.57 ± 1.02	4.23 ± 1.14	0.15, 0.54	0.001

*Note: N*, number of participants; *p*, probability value.

Abbreviations: BOP, bleeding on probing; CAL, clinical attachment level; CI, confidence interval; PD, pocket depth; PI, plaque index; SD, standard deviation.

The clinical parameters of the participants on the control side before and after the intervention are reported in Table 2. The mean BOP score decreased from baseline (75.33 ± 13.02) to the week 6 of the intervention (62.30 ± 10.73), with the reduction being statistically significant (*p* = 0.000). There was also a substantial reduction in PI score from baseline (82.86 ± 13.80) to postintervention (68.88 ± 12.38; *p* = 0.000). A statistically significant mean reduction in CAL was observed from baseline (4.88 ± 1.55) to week 6 (4.65 ± 1.69), with *p* = 0.011. The mean PD reduced from baseline (4.57 ± 1.02) to week 6 (4.23 ± 1.14), with *p* = 0.001.

**Figure 2:**
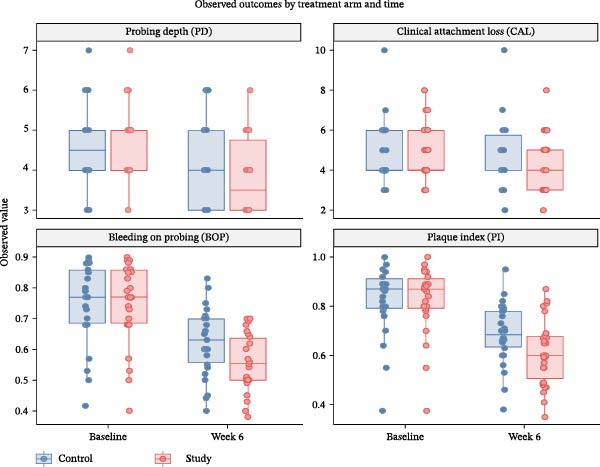
Observed periodontal outcomes in the control and study sides at baseline and 6 weeks.

A comparison of clinical parameters before and after the intervention on both the test and control sides is presented in Table 3. At baseline, no statistically significant differences were observed in BOP, PI, CAL, or PD between the test and control groups, ensuring baseline comparability. However, by the sixth week of intervention, the test side exhibited a significantly greater reduction in mean BOP score (55.92 ± 9.34) compared with the control side (62.30 ± 10.73), with *p* = 0.000. The PI score was also lower at the test side (61.23 ± 13.47) than at the control side (68.88 ± 12.38), with the difference being statistically significant (*p* = 0.000). The test side demonstrated greater improvement in mean CAL score (4.15 ± 1.43) compared with the control side (4.65 ± 1.69), with *p* = 0.001. The mean PD score also exhibited a statistically significant reduction at the test side (3.80 ± 0.93) vs. the control side (4.23 ± 1.14), with *p* = 0.000 (Figure 2).

**Table 3:** Comparisons of clinical parameters before and after the intervention in both test and control sides.

Variables	*N*	Baseline (mean ± SD)		95% CI	*p*	6^th^ week (mean ± SD)		95% CI	*p*
		Test side	Control side			Test side	Control side		
BOP	26	75.26 ± 13.19	75.33 ± 13.02	−0.06, 0.18	0.327	55.92 ± 9.34	62.30 ± 10.73	3.33, 9.43	0.000
PI	26	82.86 ± 13.80	82.86 ± 13.80	—	—	61.23 ± 13.47	68.88 ± 12.38	4.25, 11.04	0.000
CAL	26	4.84 ± 1.37	4.88 ± 1.55	−0.20, 0.28	0.746	4.15 ± 1.43	4.65 ± 1.69	0.23, 0.76	0.001
PD	26	4.53 ± 0.98	4.57 ± 1.02	0.17, 0.25	0.713	3.80 ± 0.93	4.23 ± 1.14	0.21, 0.62	0.000

*Note: N*, number of participants; *p*, probability value.

Abbreviations: BOP, bleeding on probing; CAL, clinical attachment level; CI, confidence interval; PD, pocket depth; PI, plaque index; SD, standard deviation.

### 3.1. Mixed Model Analysis

To account for the split‐mouth structure of the trial and the repeated baseline and 6‐week measurements within the same participant, a primary linear mixed‐effects model was fitted for each clinical outcome. In this model, treatment side (study vs. control), time (baseline vs. week 6), and the treatment with time interaction were entered as fixed effects, while participant‐level random effects were used to account for the correlation between repeated observations taken from the same individual. Clinically, this modeling strategy is important because each patient contributed observations from both sides of the mouth and at two time points; therefore, the measurements were not statistically independent. Within this framework, the intercept represents the estimated mean value in the control side at baseline, the study vs. control baseline term reflects whether the two sides differed before treatment, the time term reflects the degree of improvement in the control side over 6 weeks, and the interaction term represents the additional improvement observed in the study side beyond that achieved by SRP alone. Thus, the interaction term is the key estimate for judging the adjunctive clinical effect of ozone therapy.

In Table 4 of primary mixed models, for PD, the estimated baseline mean in the control side was 4.62 mm (95% CI: 4.21–5.03; *p*  < 0.001). There was no evidence of a baseline difference between study and control sides (*p* = 0.489), indicating good baseline comparability. Over 6 weeks, the control side showed a significant reduction in PD of 0.38 mm, confirming that conventional nonsurgical periodontal therapy alone produced measurable improvement. Importantly, the study side demonstrated an additional PD reduction of 0.35 mm beyond the control side (interaction estimate = −0.35 mm, 95% CI: −0.63 to −0.06; *p* = 0.019). From a clinical standpoint, this suggests that adjunctive ozone therapy conferred a further benefit in pocket reduction beyond SRP alone, although the magnitude of this additional effect was modest.

**Table 4:** Primary linear mixed‐effects model of periodontal outcomes in the split‐mouth trial.

Outcome	Term	Estimate (mean difference)	95% CI lower	95% CI upper	*p*‐Value
Probing depth (PD)	Intercept (control at baseline)	4.615	4.205	5.026	<0.001
	Study vs. control at baseline	−0.077	−0.298	0.144	0.489
	Week 6 vs. baseline in control	−0.385	−0.588	−0.182	<0.001
	Additional change in study vs. control	−0.346	−0.633	−0.059	0.019
Clinical attachment loss (CAL)	Intercept (control at baseline)	4.885	4.274	5.496	<0.001
	Study vs. control at baseline	−0.039	−0.291	0.214	0.760
	Week 6 vs. baseline in control	−0.231	−0.408	−0.053	0.012
	Additional change in study vs. control	−0.462	−0.713	−0.211	<0.001
Bleeding on probing (BOP)	Intercept (control at baseline)	0.753	0.707	0.800	<0.001
	Study vs. control at baseline	−0.001	−0.033	0.031	0.969
	Week 6 vs. baseline in control	−0.130	−0.162	−0.098	<0.001
	Additional change in study vs. control	−0.063	−0.108	−0.018	0.007
Plaque index (PI)	Intercept (control at baseline)	0.829	0.776	0.882	<0.001
	Study vs. control at baseline	0.000	−0.037	0.037	1.000
	Week 6 vs. baseline in control	−0.140	−0.177	−0.103	<0.001
	Additional change in Study vs. control	−0.077	−0.129	−0.025	0.004

For CAL, the control‐side baseline mean was 4.88 mm, and again no significant baseline difference was observed between sides. The control side improved significantly overtime, with a mean CAL reduction of 0.23 mm (95% CI: −0.41 to −0.05; *p* = 0.012). More importantly, the study side achieved an additional CAL gain of 0.46 mm compared with the control side (95% CI: −0.71 to −0.21; *p*  < 0.001). Among the modeled outcomes, this appears to be one of the strongest adjunctive effects, suggesting that ozone therapy may have contributed meaningfully to attachment improvement in addition to standard mechanical therapy. For BOP, the estimated baseline level in the control side was 0.753 (95% CI: 0.707–0.800; *p*  < 0.001), with no significant baseline difference between study and control sides. The control side showed a clear reduction overtime (estimate = −0.130, *p*  < 0.001), indicating a substantial decrease in gingival bleeding following SRP. In addition, the study side showed a further reduction of 0.063 beyond the control side. Clinically, this finding supports an added anti‐inflammatory effect of adjunctive ozone therapy, with the study side exhibiting less bleeding after treatment than would be expected from SRP alone. For PI, the control‐side baseline mean was 0.829 (95% CI: 0.776–0.882; *p*  < 0.001), and there was no baseline imbalance between sides. The control side improved significantly over time, with a reduction of 0.140. The study side showed an additional reduction of 0.077 beyond the control side. Clinically, this suggests that plaque control improved in both groups after treatment, but the adjunctive ozone‐treated side experienced a significantly greater reduction, which may reflect either enhanced local decontamination or improved periodontal healing conditions (Table 4).

In Table 5, the secondary analysis used an adjusted linear mixed‐effects model, which extends the primary model by accounting not only for the split‐mouth and repeated‐measures structure of the data, but also for relevant patient‐level clinical factors, including age, periodontitis stage, smoking duration, and number of cigarettes smoked per day. As in the primary model, the most important term is the treatment × time interaction, reported here as the additional change in study vs. control, because it represents the extra improvement attributable to adjunctive ozone therapy beyond the effect of SRP alone. For PD, the control side showed a significant reduction overtime of 0.38 mm, and the study side demonstrated an additional reduction of 0.35 mm beyond the control side, indicating that the adjunctive benefit of ozone remained significant even after adjustment. Among the covariates, older age was associated with greater PD (*β* = 0.049 mm per year, 95% CI: 0.013–0.086; *p* = 0.011), Stage III disease was associated with approximately 1.00 mm higher PD than Stage II (*p*  < 0.001), and greater cigarette consumption was also associated with higher PD (*β* = 0.016 mm per cigarette/day, 95% CI: 0.004–0.028; *p* = 0.013), whereas smoking duration was not significant (Table 5).

**Table 5:** Secondary adjusted linear mixed‐effects model of periodontal outcomes after controlling for age, disease stage, and smoking‐related variables.

Outcome	Term	Estimate (mean difference)	95% CI lower	95% CI upper	*p*‐Value
Probing depth (PD)	Intercept (control at baseline)	2.230	1.332	3.128	<0.001
	Study vs. control at baseline	−0.077	−0.298	0.144	0.489
	Week 6 vs. baseline in control	−0.385	−0.588	−0.182	<0.001
	Age (years)	0.049	0.013	0.086	0.011
	Stage III vs. Stage II	1.001	0.623	1.380	<0.001
	Smoking duration (years)	−0.005	−0.042	0.032	0.775
	Number of cigarettes/day	0.016	0.004	0.028	0.013
	Additional change in study vs. control	−0.346	−0.633	−0.059	0.019
Clinical attachment loss (CAL)	Intercept (control at baseline)	2.365	0.247	4.483	0.030
	Study vs. control at baseline	−0.039	−0.291	0.214	0.760
	Week 6 vs. baseline in control	−0.231	−0.408	−0.053	0.012
	Age (years)	0.037	−0.051	0.124	0.394
	Stage III vs. Stage II	1.690	0.791	2.589	<0.001
	Smoking duration (years)	0.023	−0.064	0.111	0.589
	Number of cigarettes/day	0.009	−0.020	0.039	0.521
	Additional change in study vs. control	−0.462	−0.713	−0.211	<0.001
Bleeding on probing (BOP)	Intercept (control at baseline)	0.752	0.466	1.038	<0.001
	Study vs. control at baseline	−0.001	−0.033	0.031	0.969
	Week 6 vs. baseline in control	−0.130	−0.162	−0.098	<0.001
	Age (years)	−0.001	−0.013	0.011	0.826
	Stage III vs. Stage II	0.071	−0.050	0.193	0.236
	Smoking duration (years)	0.002	−0.010	0.013	0.792
	Number of cigarettes/day	−0.001	−0.004	0.004	0.814
	Additional change in study vs. control	−0.063	−0.108	−0.018	0.007
Plaque index (PI)	Intercept (control at baseline)	0.845	0.535	1.155	<0.001
	Study vs. control at baseline	0.000	−0.037	0.037	1.000
	Week 6 vs. baseline in control	−0.140	−0.177	−0.103	<0.001
	Age (years)	−0.003	−0.016	0.010	0.620
	Stage III vs. Stage II	0.100	−0.032	0.232	0.129
	Smoking duration (years)	0.003	−0.010	0.016	0.609
	Number of cigarettes/day	0.000	−0.004	0.005	0.895
	Additional change in study vs. control	−0.077	−0.129	−0.025	0.004

**Figure 3:**
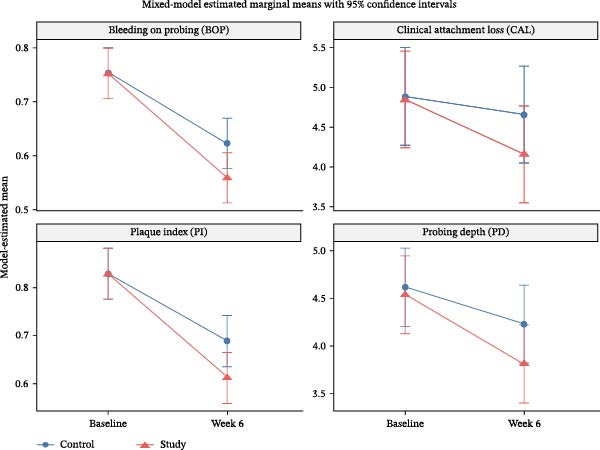
Estimated marginal means from the mixed‐effects model for periodontal outcomes in the control and study sides at baseline and 6 weeks.

For CAL, the control side improved by 0.23 mm overtime, while the study side showed an additional gain of 0.46 mm compared with control (*p*  < 0.001), again supporting a persistent adjunctive effect of ozone. The main clinical predictor of worse CAL was Stage III periodontitis, which was associated with 1.69 mm greater CAL than Stage II (*p*  < 0.001), while age, smoking duration, and number of cigarettes/day were not significant after adjustment. For the inflammatory and hygiene‐related outcomes, the pattern remained consistent. In BOP, the control side improved significantly overtime by 0.130, and the study side showed an additional reduction of 0.063. In PI, the control side improved by 0.140 (*p*  < 0.001), with a further reduction of 0.077 in the study side. None of the adjustment covariates were significantly associated with BOP or PI. There were still no significant baseline differences between study and control sides across all outcomes, which supports baseline comparability within the split‐mouth design (Figure 3). Overall, the adjusted model confirms the primary findings: both sides improved after treatment, but the ozone‐treated side showed significantly greater additional improvement in PD, CAL, BOP, and PI. Clinically, this suggests that the adjunctive benefit of ozone therapy is robust with the clearest structural effects seen for PD and CAL, and the clearest inflammatory/hygiene effects seen for BOP and PI (Table 5 and Figure 3).

Table 6 shows model fitmetrics using AIC and BIC for comparative fit, together with marginal *R* ^2^ and conditional *R* ^2^, where marginal *R* ^2^ reflects the proportion of variance explained by the fixed effects alone and conditional *R* ^2^ reflects the variance explained by the full model, including both fixed and random effects. For PD, the secondary adjusted mixed model showed clearly better fit than the primary model, with substantially lower AIC/BIC (176.56/205.65 vs. 200.76/219.27) and a much higher marginal *R* ^2^ (0.8017 vs. 0.0882), while conditional *R* ^2^ remained similarly high (0.8845 vs. 0.8851), indicating that the improved fit mainly came from the added clinical covariates rather than changes in the random‐effects structure (Table 6).

**Table 6:** Model fit indices for the primary and secondary mixed‐effects models.

Outcome	Model	Random structure of the model	AIC	BIC	Marginal *R* ^2^	Conditional *R* ^2^
Probing depth (PD)	Primary mixed model	Patient and mouth‐side clustering	200.76	219.27	0.088	0.885
Clinical attachment loss (CAL)	Primary mixed model	Patient and mouth‐side clustering	220.95	239.46	0.036	0.958
Bleeding on probing (BOP)	Primary mixed model	Patient and mouth‐side clustering	−194.77	−176.25	0.682	0.813
Plaque index (PI)	Primary mixed model	Patient clustering only	−168.78	−152.91	0.328	0.834
Probing depth (PD)	Secondary adjusted mixed model	Adjusted; patient and mouth‐side clustering	176.56	205.65	0.802	0.885
Clinical attachment loss (CAL)	Secondary adjusted mixed model	Adjusted; patient and mouth‐side clustering	211.99	241.08	0.684	0.959
Bleeding on probing (BOP)	Secondary adjusted mixed model	Adjusted; patient clustering only	−157.88	−131.44	0.367	0.853
Plaque index (PI)	Secondary adjusted mixed model	Adjusted; patient clustering only	−133.17	−106.73	0.401	0.846

For CAL, the secondary model also showed a lower AIC (211.99 vs. 220.95) and a markedly higher marginal *R* ^2^ (0.6836 vs. 0.0357), with conditional *R* ^2^ remaining almost identical (0.9586 vs. 0.9577); although the BIC was slightly higher in the adjusted model (241.08 vs. 239.46), the overall pattern still suggests better explanatory performance after adjustment. Overall, where direct comparison was possible, the secondary adjusted mixed model is preferable, particularly for PD and CAL, because it provides better fit and much greater explanatory power while preserving the same core treatment effect (Table 6).

## 4. Discussion

The findings of the present study demonstrated that ozone therapy used as an adjunct to SRP significantly improved periodontal clinical parameters compared with SRP alone in smokers with periodontitis. These results are consistent with those reported by Alsakr et al. [28], who evaluated adjunctive ozone therapy in periodontal treatment at our institution. However, the present study is an independent investigation with a different study population and design, focusing specifically on smokers with Stages II and III periodontitis using a prospective split‐mouth clinical design. In addition, a previous study reported a highly significant improvement in clinical parameters and bacterial counts in quadrants treated with SRP combined with ozone application compared with SRP alone in patients with aggressive periodontitis [29]. One of the primary goals of periodontal treatment is to eliminate biofilms using nonsurgical mechanical debridement, such as SRP. However, achieving complete removal of subgingival calculi through SRP alone is challenging, particularly when pocket depths exceed 5 mm [30]; as such, there is an increasing need for an adjunctive agent [31]. In our study, CAL was significantly improved in the quadrants treated with ozone. The PD decreased substantially, from 4.53 to 3.80 mm by the sixth week of treatment. Although the differences observed between the test and control sites were relatively modest (~0.5–0.7 mm), these improvements may still be clinically relevant. In nonsurgical periodontal therapy, reductions in PD and gains in clinical attachment level, even when small, may contribute to improved periodontal stability and reduced disease progression. Previous studies have shown that SRP typically results in PD reductions of approximately 1–2 mm depending on baseline pocket depth, and adjunctive therapies may provide additional incremental benefits. Therefore, the additional improvements observed with adjunctive ozone therapy in the present study may represent a clinically meaningful enhancement of treatment outcomes, particularly in smokers who are known to demonstrate a reduced response to periodontal therapy [32]. Similar findings were observed by Kshitish and Laxman [33] and Katti and Chava [34], who demonstrated that irrigation with ozonated water led to a reduction in PD even in 6 mm pocket within 4 weeks. This reduction could be attributed to the antimicrobial and anti‐inflammatory properties of ozone, which promote healing and regeneration of the long junctional epithelium [35]. Recent evidence has further supported the adjunctive role of ozone therapy in periodontal treatment. A recent meta‐analysis by Liu et al. [35] reported that ozone therapy combined with SRP may provide additional clinical benefits in the management of periodontitis. Similarly, recent randomized clinical trials and systematic reviews have highlighted the antimicrobial and anti‐inflammatory potential of ozone‐based therapies in periodontal treatment [36, 37].

Smoking is an independent risk factor for the initiation and progression of periodontal disease. This leads to the loss of periodontal attachment and alveolar bone density as well as an increase in the number and depth of periodontal pockets. Moderate to severe gingival inflammation (Stages II and III according to the new classification in 2017) is more pronounced in smokers than in nonsmokers [7]. Therefore, the removal of microbial dental plaque is more critical in smokers. In our study, 26 smokers were diagnosed with Stage II (53.8%) or Stage III (46.2%) periodontitis; at the end of the sixth week, quadrants treated with SRP combined with ozonated water exhibited significant improvement in clinical parameters. This finding aligns with that of Talmaç and Çalişir [24] and can be attributed to the strong antimicrobial and anti‐inflammatory effects of ozone. Furthermore, the ability of ozone to stimulate the circulatory system and modify the immunological response makes it an ideal therapeutic agent for treating oral pathologies and periodontal diseases in smokers. This explain The results of our study which revealed a statistically significant improvement in BOP in the sixth week of postintervention assessment on the test side. This finding aligns with previous studies, such as those by Ameyaroy et al. [38] and Ramirez‐Peña et al. [39], who reported similar improvements after ozone therapy. Menabde et al. [40] also observed a reduction in gingival inflammation and restoration of the normal state of the gingiva within just 3 days of ozone therapy. Ozone is a powerful oxidizing agent with high antimicrobial efficacy against oral pathogens. The reduction in bleeding upon probing and the improvement in gingival inflammation could be attributed to the anti‐inflammatory properties of ozone. These properties help modulate the inflammatory response in periodontal tissues, thereby facilitating faster healing and reducing clinical signs of inflammation. However, our findings differ from those of Hayakumo et al. [41], who reported that adjunctive therapy with ozone did not result in any additional positive effects on the clinical parameters studied compared with SRP treatment alone in patients diagnosed with chronic periodontitis.

Regarding the PI, a statistically significant reduction in PI scores was observed from baseline to the sixth week on both the test and control sides. However, SRP combined with ozone therapy resulted in a greater reduction in PI scores, from 82.86 ± 13.80 to 61.23 ± 13.47 by week 6. Ozone, a potent oxidizer, is well‐known for its ability to destroy bacteria, fungi, and viruses in both gaseous and aqueous phases by targeting cell walls and cytoplasmic membranes. Previous studies [42–44] have demonstrated that nonsurgical mechanical therapies, such as SRP, can reduce the prevalence and levels of key periodontal pathogens. In contrast, in a previous study, SRP alone achieved statistically significant reductions in periodontal pathogens in the first month after treatment, whereas SRP with ozone had the same effect after 3 months of treatment [43]. Nagayoshi et al. [44] reported that ozonated water (0.5–4 mg/L) is highly potent at killing both gram‐positive and gram‐negative oral bacteria. Similarly, Bezrukova et al. [45] found that both gaseous and aqueous ozone therapies inhibited the growth of *Aggregatibacter actinomycetemcomitans* (Aa), *Tannerella forsythia* (Tf), *Treponema denticola* (Td), *Porphyromonas gingivalis* (Pg), and *Prevotella intermedia* (Pi).

All participants in the present study were tobacco users diagnosed with moderate‐to‐severe periodontitis and were advised to continue their regular oral hygiene practices and diet. This helped us evaluate the effectiveness of ozonated water without any confounding factors. A split‐mouth design was used to eliminate patient‐specific factors, enabling the comparison of the two treatment procedures under similar conditions. Although the study duration was 6 weeks, it demonstrated a greater improvement in clinical parameters without any adverse effects from ozonated water.

Chlorhexidine is widely recognized for its antimicrobial action and is considered to be the gold standard among antiplaque chemical agents due to its high substantivity, which ensures prolonged antimicrobial activity [33]. Despite its efficacy, chlorhexidine is associated with several adverse effects. These include irritation of the mucous membranes, impaired wound healing, pigmentation changes in the teeth and tongue, and alterations in taste perception [46]. Ozone may represent a valid alternative to chlorhexidine and other adjunct solutions in periodontal treatment [31]. Due to its ease of application, noninvasive approach, absence of discomfort, and high level of patient acceptability, ozonated water presents a promising adjunctive option for SRP in clinical periodontal therapy.

It should also be noted that the follow‐up period of 6 weeks represents a relatively short time frame for evaluating periodontal outcomes, particularly clinical attachment level changes. Therefore, the improvements observed in the present study likely reflect short‐term periodontal healing following nonsurgical therapy rather than long‐term periodontal stability. Longer observation periods are generally required to determine the stability of periodontal treatment outcomes and attachment level changes following periodontal therapy[47, 48]

The mixed‐model results show a highly consistent pattern across all four clinical parameters. First, the absence of significant baseline side differences for PD, CAL, BOP, and PI supports the internal validity of the split‐mouth comparison and indicates that the two sides were comparable before intervention. Second, both sides improved overtime, confirming the expected therapeutic effect of SRP. Third, and most importantly, the significant negative interaction terms across all outcomes indicate that the study side improved more than the control side, demonstrating an adjunctive benefit of ozone therapy. Clinically, the added effect was most pronounced for CAL and PD, which are key indicators of periodontal healing and treatment response, while additional favorable reductions were also observed in inflammatory bleeding and plaque accumulation.

The secondary analysis used an adjusted linear mixed‐effects model, which extends the primary model by accounting not only for the split‐mouth and repeated‐measures structure of the data, but also for relevant patient‐level clinical factors, including age, periodontitis stage, smoking duration, and number of cigarettes smoked per day. In this framework, the fixed effects estimate the average clinical associations of treatment side, time, and covariates across the study population, whereas the random effects account for the fact that repeated observations from the same patient are correlated and, therefore, should not be treated as independent. Clinically, this means the model estimates the adjunctive effect of ozone therapy after controlling for differences in patient severity and smoking exposure, while still respecting the within‐patient nature of the split‐mouth design. The primary and secondary mixed‐model analysis supports the conclusion that adjunctive ozone therapy provided measurable short‐term clinical benefit beyond SRP alone in smokers with Stages II and III periodontitis. Overall, where direct comparison was possible, the secondary adjusted mixed model is preferable, particularly for PD and CAL, because it provides better fit and much greater explanatory power while preserving the same core treatment effect.

The present study has several limitations that should be considered when interpreting the findings. Although the split‐mouth design minimized interindividual variability, it may not be ideal for evaluating topical interventions such as ozone therapy due to the potential for cross‐contamination between test and control sites. In addition, the study included a relatively small sample size and was conducted over a short follow‐up period with a single application of ozone therapy, which limits the assessment of long‐term treatment outcomes. The study was also conducted at a single center, which may affect the generalizability of the findings. Furthermore, only tobacco users with Stages II and III periodontitis were included, and therefore, the results may not be fully generalizable to other populations, such as nonsmokers or users of other tobacco products including electronic cigarettes. Another limitation is the absence of microbiological or inflammatory biomarker analysis, such as evaluation of periodontal pathogens in gingival crevicular fluid, which could provide additional insight into the biological effects of ozone therapy. Future studies with larger sample sizes, longer follow‐up periods, repeated ozone applications, and microbiological or biomarker assessments are recommended to further evaluate the effectiveness of ozone therapy. Additionally, comparisons between ozone therapy and other adjunctive periodontal treatments, such as antiseptic mouthwashes, may provide further clinical insight.

## 5. Conclusions

Considering the limitations of this study, we conclude that freshly prepared ozonated water has the potential to be an effective adjunct for managing periodontitis among smokers. Improvements in CAL, reduction in PD, plaque scores, and BOP suggest the positive impact of ozonated water when used as an adjunct to nonsurgical periodontal therapy. Given its simplicity, noninvasive nature, pain‐free application, and good patient tolerance, ozonated water may be a viable adjunct to SRP in clinical settings. Nevertheless, further research is required to evaluate its microbiological and biochemical efficacy as well as to determine the optimal concentration and application frequency.

## Author Contributions

Hanadi Ghurmallah Alzahrani, Banna Alnufaiy, Hassan Alkharaan, and Abdulaziz Alsakr contributed to the study design and conceptualization. Hanadi Ghurmallah Alzahrani and Khalid Gufran contributed to project supervision and methodology. Hanadi Ghurmallah Alzahrani contributed to acquiring ethical approval, formal analysis, validation, and writing the original draft. Faisal Mohammed Alharbi, Naif Salem Alanazi, and Muath Abdullah Alsuwailem contributed to data collection. Khalid Gufran and Hanadi Ghurmallah Alzahrani contributed to the review and editing of the final draft.

## Funding

This research received no external funding.

## Ethics Statement

The study was conducted in accordance with the Declaration of Helsinki and approved by the standing committee of bioethics research (SCBR) of Prince Sattam bin Abdulaziz University approved this study protocol (SCBR‐158‐2023), approved on 02‐10‐2023.

## Consent

All the participants gave consent for the publication of the data. All patients have signed the informed consent to participate in the study.

## Conflicts of Interest

The authors declare no conflicts of interest.

## Data Availability

The datasets used and/or analyzed during the current study are available from the corresponding author upon reasonable request.
